# High Pressure Laminates with Antimicrobial Properties

**DOI:** 10.3390/ma9020100

**Published:** 2016-02-06

**Authors:** Sandra Magina, Mauro D. Santos, João Ferra, Paulo Cruz, Inês Portugal, Dmitry Evtuguin

**Affiliations:** 1 CICECO–Aveiro Institute of Materials and Chemistry Department, University of Aveiro, Campus Universitário de Santiago, 3810-193 Aveiro, Portugal; smagina@ua.pt (S.M.); mdbs@ua.pt (M.D.S.); inesport@ua.pt (I.P.); 2 EuroResinas–Indústrias Químicas SA, Plataforma Industrial de Sines-Lote Industrial I, Monte Feio, 7520-064 Sines, Portugal; joao.ferra@sonaeindustria.com; 3 SONAE Indústria de Revestimentos SA (SIR), Lugar do Espido-Via Norte, Apartado 1096, 4470-177 Maia, Portugal; paulo.cruz@sonaeindustria.com

**Keywords:** PHMB, high pressure laminates, antimicrobial, bacteriostatic, bactericidal, surface, melamine-formaldehyde resin

## Abstract

High-pressure laminates (HPLs) are durable, resistant to environmental effects and good cost-benefit decorative surface composite materials with special properties tailored to meet market demand. In the present work, polyhexamethylene biguanide (PHMB) was incorporated for the first time into melamine-formaldehyde resin (MF) matrix on the outer layer of HPLs to provide them antimicrobial properties. Chemical binding of PHMB to resin matrix was detected on the surface of produced HPLs by attenuated total reflection Fourier transform infrared spectroscopy (ATR-FTIR). Antimicrobial evaluation tests were carried out on the ensuing HPLs doped with PHMB against gram-positive *Listeria innocua* and gram-negative *Escherichia coli* bacteria. The results revealed that laminates prepared with 1.0 wt % PHMB in MF resin were bacteriostatic (*i.e.*, inhibited the growth of microorganisms), whereas those prepared with 2.4 wt % PHMB in MF resin exhibited bactericidal activity (*i.e.*, inactivated the inoculated microorganisms). The results herein reported disclose a promising strategy for the production of HPLs with antimicrobial activity without affecting basic intrinsic quality parameters of composite material.

## 1. Introduction

**Figure 1:**
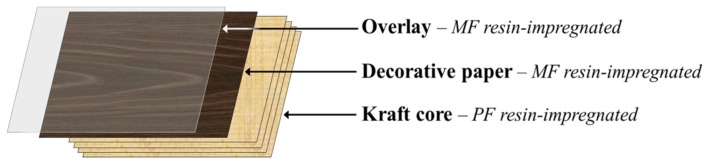
Typical assembly for a high pressure laminates build-up.

High-pressure laminates (HPLs) are multipurpose decorative materials used by the furnishing and building industries for the production of furniture, countertops, flooring and wall paneling surfaces used in different sectors, namely in residential and working environments and in public places (for instance, hospitals and schools). HPLs display high durability and special surface properties such as chemical, heat, stain and wear resistance tailored to meet market demand. Conventionally, HPLs consist of an assembly of 5 to 50 layers of kraft paper (core) saturated with phenol-formaldehyde (PF) resin, topped by a single printed or colored decorative paper layer saturated with melamine-formaldehyde (MF) resin and, in some cases, by a finishing protective translucent overlay also impregnated with MF resin (Figure 1) [1,2]. After build-up, these layers are transformed into a single rigid laminated sheet by heat and high-pressure treatments. During this curing process, the PF and MF thermosetting resins undergo a series of cross-linking reactions, thus creating strong irreversible bonds between the resin matrix and the impregnated paper layers [1]. Nowadays, laminate producers seek the development of HPLs incorporating new and combined features (*i.e*., multifunctionality) to gain competitive advantage in national and international markets.

Severe infectious diseases may be acquired not only in hospitals and other health care facilities but also in domestic environments due to (cross-)contaminations from different sources, such as infected people, animals, food and/or water [3]. Moreover, bacteria are known to persist on surfaces for months [4], so the improvement of hygienic and antimicrobial properties of laminate surfaces is of utmost importance. Current approaches to eliminate or decrease bacterial attachment on surfaces (*i.e.*, to create antibacterial surfaces) include surface coating and surface modification by physical methods (structuring, e.g., lotus leaf and shark skin effect) or chemical methods (e.g., surface polymerization, functionalization or derivatization) [5]. A vast array of chemicals exhibiting antimicrobial properties, ranging from low-molecular weight organic and inorganic compounds to nanoparticles or polymeric materials, has been reported as possible candidates for incorporation into melamine resins surfaces [6]. In particular, colloidal silver has been used to produce laminate wood flooring exhibiting antibacterial activity [7].

The incorporation of antibacterial agents in laminates requires information not only about their efficacy and stability after HPL build-up (hot pressing) but also about their toxicity to living organisms and the environment. For instance, quaternary ammonium salts that are frequently used as disinfectants in food-processing industries may cause respiratory, skin and eye irritation [8]. Triclosan, an antibacterial and antifungal agent used for surgical cleaning treatments may be considered an endocrine disrupter [9,10,11]. Nanoparticles have been reported to accumulate and/or pass into the circulatory and lymphatic systems, thus exhibiting toxicity to humans and animals [12,13]. In particular, silver nanoparticles, which are increasingly used as antibacterial agents in commercial products and medical devices [14], pose serious health risks to humans [15,16,17]. In contrast, poly(hexamethylene biguanide)—henceforward named PHMB—is a powerful yet safe antiseptic [18].

**Figure 2:**
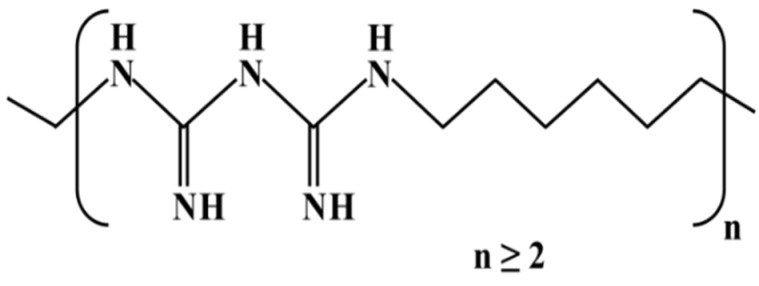
Chemical structure of poly(hexamethylene biguanide).

PHMB is a chemically stable water-soluble positively charged polymer (Figure 2) whose chemical composition was revealed in the late 1950s [19,20]. It is a reasonably priced broad-spectrum fast-acting bactericide, highly effective against gram-positive and gram-negative bacteria [21,22]. Therefore, a wide range of applications exists, for example, PHMB is used as a solid surface disinfectant, a swimming pool sanitizer, a preservative in cosmetics and textile products, and as a disinfectant in agriculture and food processing [21,22,23]. PHMB is also used in direct contact with humans with excellent tolerance and low risk, for instance, in contact lens solutions and in wound dressings to prevent or treat infections [19,24,25,26]. Notably, PHMB is effective against multidrug-resistant bacteria [19] and much more efficient than antiseptics such as chlorhexidine, PVP-iodine, triclosan, silver and sulfadiazine [26].

Being a polymer PHMB is easily processed into films [27] and, therefore, a suitable candidate for coating a resin-impregnated decorative paper surface or to be incorporated in MF resin formulations prior to paper impregnation. Following this strategy, HPLs containing PHMB were produced by different approaches, characterized by standard evaluation tests according to EN 438-2:2005 [28], and their antibacterial activity was examined using gram-positive *Listeria innocua* (*L. innocua*) and gram-negative *Escherichia coli* (*E. coli*) bacteria.

## 2. Results and Discussion

### 2.1. Characterization of HPLs Containing PHMB

**Figure 3:**
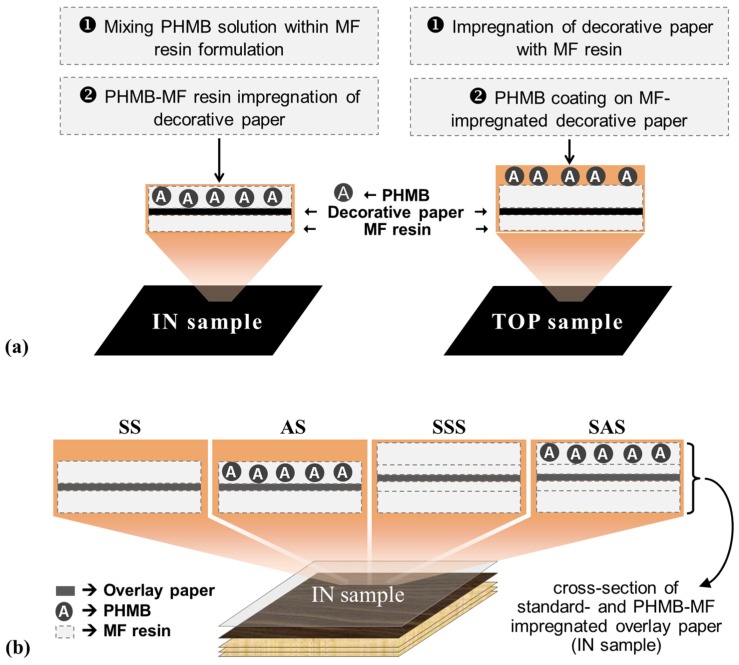
Schematic representation of HPLs containing PHMB: (**a**) decorative paper impregnation strategies; (**b**) build-up approaches for HPLs without PHMB (SS and SSS) and with PHMB (AS and SAS).

HPLs containing PHMB (1.0 and 2.4% w*/*w based on resin formulation) and conventional HPLs (0% PHMB) were produced by different impregnation strategies, as described in Experimental Section 3.2.1 and schematically depicted in Figure 3. Impregnation of the decorative paper (Figure 3a) was performed with a PHMB-MF resin formulation (IN sample) or with a conventional MF resin formulation posteriorly coated with the PHMB solution (TOP sample). Different approaches were also implemented for HPL build-up, all starting with three sheets of PF impregnated kraft paper, followed by one sheet of decorative paper, and a final overlay paper as shown in Figure 3b. The first approach consisted in a single step impregnation of the overlay paper with MF resin on both sides (SS) or MF resin on the back side and PHMB-MF resin on the top side (AS). In the second approach, in order to retain PHMB nearest to the surface, the overlay paper was impregnated twice, first with MF resin on both sides and, after an intermediate drying step, with MF resin on both sides (SSS) or with MF resin on the back side and PHMB-MF resin on the top side (SAS). In all cases the assembly was submitted to hot pressing to promote resin curing and the formation of a monolithic laminate [2].

**Figure 4:**
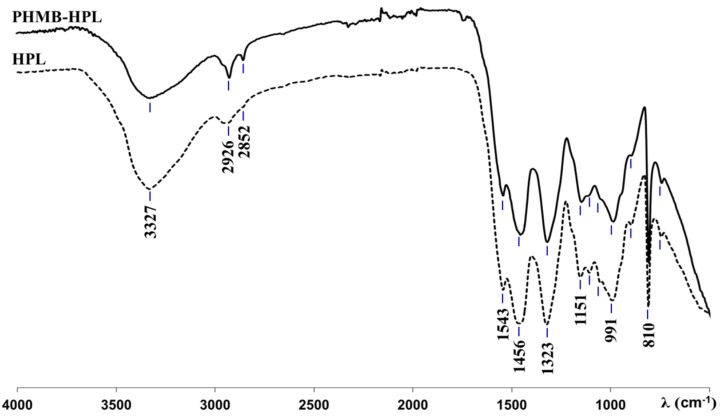
ATR-FTIR spectra of standard HPL and PHMB-HPL (2.4%(IN) SAS).

The presence of PHMB within the MF resin matrix was ascertained by ATR-FTIR analysis of conventional HPLs and those containing PHMB (HPL and PHMB-HPL, respectively). The spectra of HPL and PHMB-HPL (Figure 4) are very similar due to the common amide/imide moieties presented in melamine from MF resin and in PHMB. The similarity of aforementioned spectra is enhanced due to the eventual reaction of amide moieties in PHMB with methylol groups of MF resin, thus forming a unique polymeric network. Thus, the broad band centered at 3327 cm^−1^ was assigned to N–H stretching vibration of secondary amines, the peak at 1151 cm^−1^ corresponds to C–N vibrations, the band at 991 cm^−1^ corresponds to C–H out of plane deformation vibrations, and the peaks at 1456 and 1323 cm^−1^ relate to asymmetrical (δ_as_ CH_2_) and symmetrical (δ_s_ CH_2_) methylene C–H bending, respectively. The signals at 1109 and 1053 cm^−1^ are due to stretching vibrations of C–O and other group of MF resin, respectively, and at 810 cm^−1^ due to the triazine ring out of plan vibration from MF resin [29,30]. Nevertheless, two peaks at 2926 and 2852 cm^−1^ were detected in the PHMB-HPL spectrum, which were unnoticeable in the spectrum of HPL. These signals have been assigned to the asymmetrical (ν_as_CH_2_) and symmetrical (ν_s_CH_2_) C–H stretching vibrations, respectively, in methylene groups of hexyl moieties in PHMB [31,32]. These characteristic spectral features clearly confirm the presence of PHMB on the surface of HPLs within the MF resin matrix.

The physical and surface quality tests of AS and SAS samples of PHMB-HPL (2.4% IN) were evaluated by standard procedures (EN 438-2:2005). The results were similar for both build-up approaches and meet the requirements for HPLs except for scratching resistance (Table 1).

**Table 1:** Physical and surface quality properties of AS and SAS samples of 2.4% (IN) PHMB-HPL.

Physical Property	Test Method [28]	Required Rating	Rating *
Resistance to immersion in boiling water	EN 438-2:12	≥4	4
Resistance to water vapour	EN 438-2:14	≥4	5
Resistance to dry heat (180 °C)	EN 438-2:16	≥4	4
Resistance to impact by small diameter ball	EN 438-2:20	≥20 N	30 N
Resistance to scratching	EN 438-2:25	≥4	3
Resistance to staining (groups 1, 2 & 3)	EN 438-2:26	≥4	5

(*) 1 = Surface damage, 2 = Severe appearance alteration, 3 = Moderate change, 4 = Slight changes visible from certain angles, 5 = No change.

### 2.2. Antimicrobial activity of HPLs Containing PHMB

#### 2.2.1. Qualitative Evaluation of the Antimicrobial Activity of Decorative Paper Laminates (DPL)

It has been reported that besides phenol the formaldehyde exhibits antimicrobial activity [7] and that a formaldehyde emission from laminates is lowest when a finishing layer of melamine-impregnated paper is used [33]. Going from these considerations to rule out the influence of both phenol and formaldehyde in the antimicrobial tests, the first approach consisted in producing decorative paper laminates (DPL), comprising only the decorative paper impregnated with PHMB-MF resin, without the PF-impregnated kraft core, thus reducing the phenol and formaldehyde emissions from the newly produced laminates.

Accordingly, laminate samples containing 0.1% PHMB (both IN and TOP samples, Figure 3a) were produced without the kraft core and examined qualitatively for their antimicrobial activity using the Kirby-Bauer test. PHMB-laminates and the respective control samples did not show antimicrobial activity, *i.e.*, inhibition halos around the samples were not observed and both microorganisms (*E. coli* and *L. innocua*) exhibited growth. This situation most likely occurred because of PHMB reactions with the MF resin components during the curing period, causing loss of antimicrobial efficacy due to low content of free active biguanide PHMB moieties remaining in the laminates. In view of the results, new strategies were outlined to circumvent the problem, and a new set of samples was prepared with a higher percentage of PHMB, namely 1%(TOP), 1%(IN), and 2.4%(IN). The qualitative tests for this series of PHMB-laminates revealed antimicrobial activity (although inhibition halos were not found, microorganisms’ growth between the sample and culture medium did not occur).

#### 2.2.2. Quantitative Evaluation of the Antimicrobial Activity of DPL

**Figure 5:**
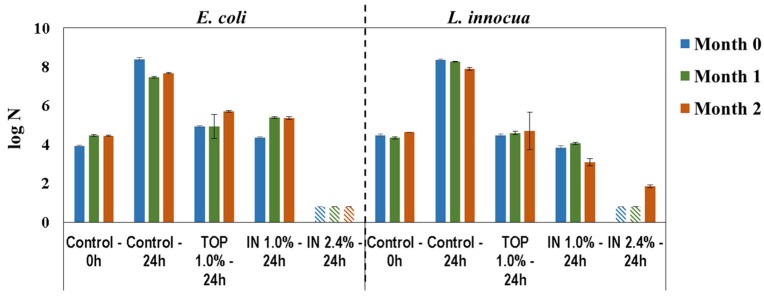
Antimicrobial activity of DPL towards *E. coli* and * L. innocua*, immediately after inoculation (0 h) and after incubation (24 h), on standard (control) and PHMB-DPL newly produced (0 month), and after 1 and 2 months storage (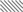 Below detection limit).

Antimicrobial activity of 1%(TOP), 1%(IN), and 2.4%(IN) PHMB-DPL was evaluated periodically during a 2-month period, using the quantitative method adapted from ISO 22196 and described in Experimental Section 3.2.2. The results displayed in Figure 5 represent the log number of viable cell per unit area, N (viable bacteria/cm^2^—see Equation (1)) immediately after inoculation (0 h) and after incubation (24 h) for the test (PHMB-DPL) and control (DPL) samples. In general, all samples exhibited antimicrobial properties for *E. coli* and *L. innocua* since in their presence both bacteria were inactivated (*i.e.*, log N lower than for the 0 h control) or their growth inhibited (*i.e.*, log N lower than for the 24 h control). In particular, 1.0%(IN) and 1.0%(TOP) PHMB-surfaces are bacteriostatic (inhibitors of bacteria growth) and the 2.4%(IN) PHMB-surface is bactericidal (capable of killing bacteria) [34]. It was noteworthy that, for the later samples, the microbial load after 24 h was lower than the detection limit (viz. log N < 0.80).

The results for antimicrobial activity after 0, 1 and 2 months of storage (Figure 5) were similar, without evidence of chances in bacteriostatic efficacy for the 1.0%(TOP) sample. However, for the 1.0%(IN) sample bacteriostatic efficacy for *E. coli* diminished (higher log N), although for *L. innocua* a small bactericidal effect was noted after 2 months *i.e.*, lower microbial load than the one inoculated (3.10 ± 0.19 *vs.* 4.63 ± 0.01 log N, respectively). Finally, for the 2.4%(IN) PHMB-DPL bactericidal activity was preserved throughout storage period (log N below the detection limit) except for the results with *L. innocua* after the 2nd month (1.85 ± 0.08 log N).

Considering the satisfactory results obtained for the 2.4%(IN) sample in terms of antimicrobial activity, it was decided that decorative paper would henceforth be impregnated using the IN mode, *i.e.*, impregnation with a PHMB-MF resin formulation (see Figure 3a).

#### 2.2.3. Quantitative Evaluation of Antimicrobial Activity of PHMB-HPLs

**Figure 6:**
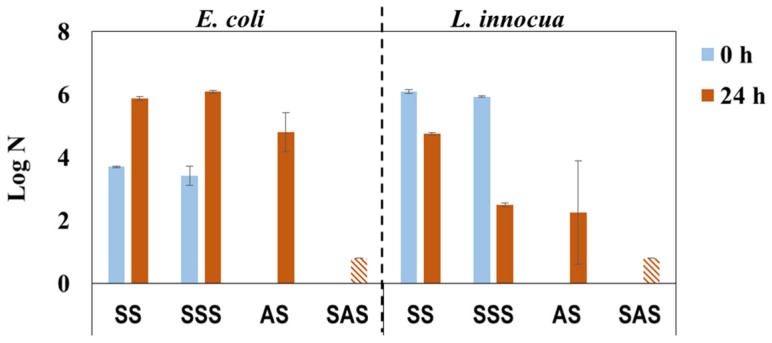
Antimicrobial activity of PHMB-HPLs prepared with two different approaches, against *E. coli* and *L. innocua* just after inoculation (0 h) and after incubation for 24 h (SS and SSS are the control samples for AS and SAS, respectively; 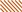, Below detection limit).

The laboratory HPLs were produced similarly to industrial HPLs with PF-impregnated kraft core using decorative paper impregnated with PHMB-MF resin (IN mode) and different approaches for build-up and overlay paper impregnation (see Figure 3b). The overlay paper is of low grammage (22 g/m^2^) and highly porous paper that becomes transparent after impregnation to unveil the appearance of the decor paper. However, these features most likely create passages for PHMB, thus lowering its concentration on the HPL surface and therefore diminishing the antimicrobial effect. The results from antimicrobial activity tests (Figure 6) reveal that the single-step impregnation approach confers some bactericidal efficacy (lower log N for AS in comparison with the SS control after 24 h incubation), thus confirming the previous reasoning. As envisioned, the two-step impregnation strategy confers significant bactericidal efficiency (log N bellow detection limit) to SAS samples.

Regarding incubated control samples (SS and SSS), a different behavior was observed for antimicrobial activity towards *E. coli* and *L. innocua*. In fact, the results for *E. coli* were similar, almost equal in both control samples (0 and 24 h), whereas the results obtained with *L. innocua* were notably different with the SSS control sample, exhibiting a lower value after incubation (24 h) than immediately after inoculation (0 h) (Figure 6). This can be related to the bactericidal effect of formaldehyde and phenol [7], which could have been present in the kraft paper used for HPL build-up, and to the lower resistance of *L. innocua* (when compared to *E. coli*) once it is recognized that gram-negative bacteria generally have a greater resistance than gram-positive bacteria [35,36,37].

**Figure 7:**
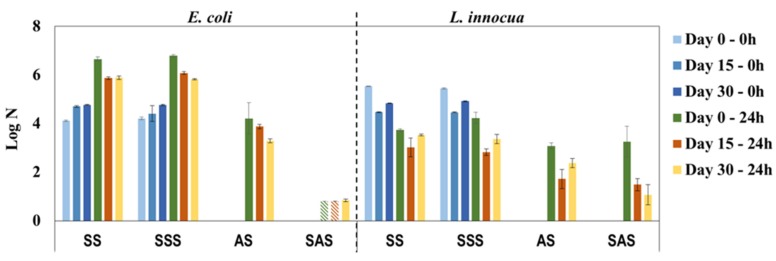
Antimicrobial properties of PHMB-HPLs produced by two different methods, against *E. coli* and *L. innocua* over a period of 30 days simulating daily use (SS and SSS are control samples for AS and SAS, respectively; 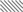, Below detection limit).

To evaluate the influence of daily use on the antimicrobial properties of PHMB-HPL surfaces, the same sample was periodically (every fortnight) cleaned with a 70% ethanol solution and analyzed over a period of 30 days. The antimicrobial activity results shown in Figure 7 reveal a constant bactericidal effect of AS and SAS samples, especially against *E. coli*, with a remarkable consistent performance of SAS during the 30-day period (log N below or close to the detection limit). The activity results against *L. innocua* present an unusual behavior with the AS and SAS samples, presenting almost no antimicrobial activity at day 0 (similar values for AS, SAS, and the respective control samples) and considerable antimicrobial efficacy for the same samples after 15 and 30 days. This discrepancy is apparently associated with unaccountable experimental error, since for the previous results (Figure 6) the log N values for SAS samples were below the detection limit for both bacteria. The facts that antimicrobial properties of PHMB-HPLs after cleaning with 70% ethanol solution were maintained and that there was no leaching of PHMB from the composite material with water (UV-Vis control of washouts after 24 h contact with composite surface) confirmed the eventual binding of this antimicrobial agent in the MF resin matrix.

**Figure 8:**
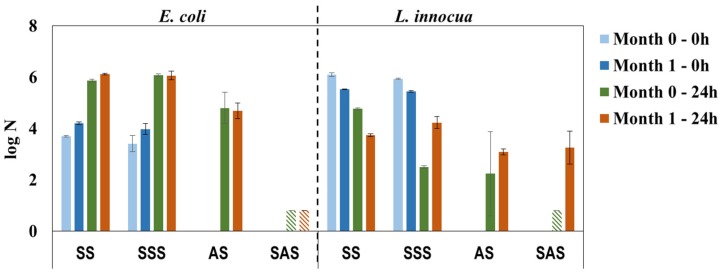
Antimicrobial properties against *E. coli* and *L. innocua* over a period of 30 days, simulating storage of PHMB-HPLs prepared by two different methods (SS and SSS are control samples for AS and SAS, respectively; 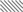, Below detection limit).

The antimicrobial properties of HPL samples recently prepared (0 month) or after storage (1 month) in bulk were practically constant against *E. coli* and confirm the bactericidal properties of SAS samples (Figure 8). For *L. innocua* the results present high standard deviation, in particular for the AS sample; nonetheless, the low value of log N (below detection limit) for the recently prepared SAS sample confirms its efficacy and pinpoints the abovementioned experimental error (*L. innocua*/Day 0/SAS sample, Figure 6 *vs.* Figure 7). It is noteworthy that storage influences the antimicrobial properties against *L. innocua* in accordance with the previous discussion about the bactericidal effect of formaldehyde/phenol [7] and the lower resistance of *L. innocua* when compared to *E. coli* [35,36,37]. In general, the results of this study reveal a reliable methodology for the preparation of HPLs with bactericidal properties.

## 3. Experimental Section

### 3.1. Materials

Black-colored decorative paper sheets (20 cm × 30 cm) and MF resin formulation were supplied by SONAE Indústria de Revestimentos (Maia, Portugal). An aqueous solution of PHMB (20% w/v) was purchased from Cymit Quimica SL (Barcelona, Spain). All of the other chemicals and reagents used for HPL production were of analytical grade.

Gram-positive bacteria Listeria innocua (L. innocua, ATCC 33090) and gram-negative bacteria *Escherichia coli* (*E. coli*, ATCC 25922) were provided by Liofilchem (Italy) and Oxoid (UK), respectively. The nutrient broth (NB) was purchased from VWR—Prolabo Chemicals (Belgium). Casein peptone lecithin polysorbate broth and the culture medium plate count agar (PCA) were purchased from Liofilchem (Italy). Mueller Hinton Agar broth (MH) was purchased from Oxoid (England). Polysorbate 20 (Tween 20) was purchased from Acros Organics (Belgium). Phosphate buffered saline (PBS) tablets were purchased from Fisher Scientific (Pittsburgh, PA, USA).

### 3.2. Methods

#### 3.2.1. Production and Characterization of HPLs

For HPL production, a stack of three layers of PF impregnated kraft paper (supplied by SIR) was topped by a sheet of MF-impregnated decorative paper and a final overlay paper (Figure 1). Decorative paper sheets were impregnated manually using two different modes: IN samples correspond to impregnation with MF resin formulation containing different loads of PHMB (0, 1.0 and 2.4% w/w resin formulation); TOP samples correspond to impregnation with MF resin followed by coating with PHMB (1.0 and 2.4% w/w based on resin formulation). Overlay paper sheets were impregnated in a single-step procedure, namely with MF resin on both sides (SS samples) or with MF resin on the back side and PHMB-MF resin on the top side (AS samples), or in a two-step procedure. The latter consisted in impregnation with standard MF resin on both sides, then an intermediate drying stage (1 min at 105 °C) followed by impregnation with MF resin on both sides (SSS samples) or with MF resin on the back side and PHMB-MF resin on the top side (SAS samples). Finally, the assembly was submitted to hot pressing (5.5 to 10.3 MPa and 120–150 °C, during 15–25 min) for resin curing [2]. The ensuing HPLs were characterized by standard procedures (EN 438–2:2005 [28]) in the laboratory of Associação Rede de Competência em Polímeros (ARCP-Porto, Portugal).

Fourier Transform Infrared (FTIR) spectroscopy was carried out on a Bruker Optics Tensor 27 coupled with a universal Attenuated Total Reflectance (ATR) Golden Gate sampling accessory. FTIR data were obtained from 4000 to 500 cm^−1^ with a 4 cm^−1^ resolution in transmittance (512 scans).

#### 3.2.2. Evaluation of Antimicrobial Activity of PHMB-HPLs

PHMB-HPLs were assessed by qualitative and quantitative tests for antimicrobial activity towards gram-negative *E. coli* and gram-positive *L. innocua* using two different methodologies to simulate storage (new samples were tested each month) and daily usage (the same samples were tested every fortnight). For storage simulation, the samples were sterilized at 121 °C) (15 min) before each test. For daily use simulation, the samples were cleaned with a 70% ethanol solution. The tests were all performed in triplicate using HPLs without PHMB as control samples.

**Figure 9:**
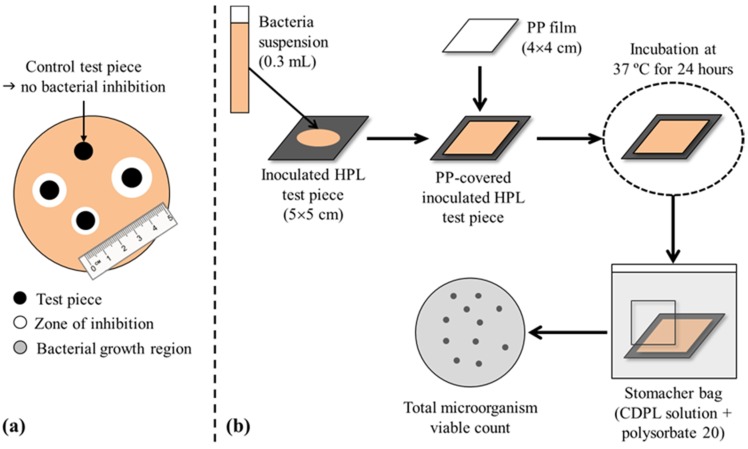
Schematic representation of the antimicrobial evaluation tests: (**a**) qualitative (disk diffusion method); (**b**) quantitative [40,41].

The qualitative antimicrobial tests were based on the disk diffusion method (the Kirby-Bauer test, Figure 9a) to determine the sensitivity/resistance of the microorganisms to PHMB in the samples. Test (PHMB-HPL) and control (HPL) samples were cut to small circular pieces (6 mm diameter), sterilized, placed on Petri-plates previously filled with MH agar, inoculated with the target microorganism, and incubated at 37 ± 1 °C) for 24 h. After incubation, the plates were inspected to verify the presence/absence of microorganism around the discs samples (inhibition halos), *i.e.*, an indirect measurement of antimicrobial properties of the HPLs [38,39].

The quantitative tests for antimicrobial activity evaluation, adapted from ISO 22196 [40] and JIS Z 2801 [41] standards, consisted in the direct inoculation of the microorganism on the samples surface (Figure 9b). Test (PHMB-HPL) and control (HPL) samples were cut in squares (50 × 50 mm^2^), sterilized at 121 °C) (15 min) or cleaned with a 70% ethanol solution (for sample reuse), and inoculated with the microorganism (0.3 mL suspension, NB diluted 500 times, with a final concentration ranging from ca 2.5 × 10^4^ to 2.5 × 10^5^ CFU/mL (colony forming units/mL). The samples (with inoculum) were covered with a sterile square-shaped polypropylene film (40 × 40 mm^2^) in order to spread the inoculum in the test specimen. The samples were analyzed (in triplicate) immediately after inoculation (0 h) and 24 h after incubation at 37 ± 1 °C) inside a petri dish lid covered with an inverted agar filled petri dish bottom and sealed with paraffin tape [42].

The microbial analysis of the samples (after inoculation and after incubation), included washing the polypropylene film with a casein peptone lecithin polysorbate broth solution and polysorbate 20 in a *stomacher* bag for 2 min using a Stomacher 80 (Seward Ldt, UK). The washing effluents were decimal diluted with a PBS solution, plated in PCA-filled plates (in duplicate), and incubated at 37 ± 1 °C) during 36–48 h (*L. innocua*) or 24 h (*E. coli*). After incubation, the number of microorganisms was determined by:

(1) $$N=\frac{C\times D\times V}{A}$$

where *N* is the number of viable bacteria per cm^2^ of sample, *C* is the colony count (mean of replicates performed in microbiology), *D* is the PBS dilution factor, *V* is the volume (cm^3^) of casein peptone lecithin polysorbate broth solution, and *A* is the polypropylene film surface area (cm^2^).

## 4. Conclusions

HPLs containing PHMB in the outer layer of composite were laboratory-made and characterized according to standard procedures, and their antimicrobial activity against *L. innocua* and *E*. *coli* was evaluated. All examined HPLs revealed bacteriostatic or bactericidal behavior when the MF resin used for the impregnation of decorative/overlay papers in the outer layer was doped with 1.0% or 2.4% of PHMB, respectively. This antimicrobial activity maintained at least for 2 months after the HPLs production, thus confirming the acquired properties during storage of composite material. At the same time, the tests simulating daily usage (cleaning with 70% ethanol every fortnight) demonstrated the permanence of antimicrobial properties for a period of at least one month. Although additional tests are required to verify the efficiency and durability of antimicrobial properties of novel HPLs, these results reveal PHMB as a promising additive for the sustainable production of laminates with antimicrobial activity.
